# Cook-Ed^TM^: A Model for Planning, Implementing and Evaluating Cooking Programs to Improve Diet and Health

**DOI:** 10.3390/nu12072011

**Published:** 2020-07-06

**Authors:** Roberta C. Asher, Tammie Jakstas, Julia A. Wolfson, Anna J. Rose, Tamara Bucher, Fiona Lavelle, Moira Dean, Kerith Duncanson, Beth Innes, Tracy Burrows, Clare E. Collins, Vanessa A. Shrewsbury

**Affiliations:** 1 Priority Research Centre for Physical Activity and Nutrition, The University of Newcastle, Callaghan, NSW 2308, Australia; roberta.asher@uon.edu.au (R.C.A.); tammie.jakstas@newcastle.edu.au (T.J.); tamara.bucher@newcastle.edu.au (T.B.); kerith.duncanson@newcastle.edu.au (K.D.); tracy.burrows@newcastle.edu.au (T.B.); clare.collins@newcastle.edu.au (C.E.C.); 2 School of Health Sciences, Faculty of Health and Medicine, The University of Newcastle, Callaghan, NSW 2308, Australia; anna.rose@newcastle.edu.au (A.J.R.); moira.dean@qub.ac.uk (M.D.); 3 Department of Health Management and Policy and Department of Nutritional Sciences, University of Michigan School of Public Health, Ann Arbor, MI 48109, USA; jwolfson@umich.edu; 4 Priority Research Centre for Health Behaviour, Faculty of Health and Medicine, The University of Newcastle, Callaghan, NSW 2308, Australia; 5 School of Environmental and Life Science, Faculty of Science, The University of Newcastle, Callaghan, NSW 2308, Australia; 6 Institute for Global Food Security, School of Biological Sciences, Queen’s University Belfast, Belfast BT9 5DL, UK; flavelle01@qub.ac.uk; 7 Challenge Community Services, Wickham, NSW 2293, Australia; beth.innes@challengecommunity.org.au

**Keywords:** cooking skills, food skills, cooking education, model, diet quality

## Abstract

Domestic cooking education programs are typically designed to improve an individual’s food and cooking skills, although not necessarily diet quality. Currently, there are no comprehensive models to guide the planning, implementation and evaluation of domestic cooking education programs that focus on improving diet and health. Our aim was to address this through development of the Cooking Education (“Cook-Ed^TM^”) model, using the PRECEDE-PROCEED model as the underlying Cook-Ed^TM^ framework. A review of the food and cooking skills education literature informed the content of the Cook-Ed^TM^ model. Cook-Ed^TM^ was critiqued by experts in consumer behaviour, cooking and nutrition education research and education until consensus on model content and format was reached. Cook-Ed^TM^ leads cooking program developers through eight distinct stages, engaging key stakeholders in a co-design process from the outset to tailor programs to address the need of individuals and inform the development of program content, program delivery, and evaluation. A Cook-Ed^TM^ scenario applied in practice is described. The proposed Cook-Ed^TM^ model has potential to be adapted for use in domestic cooking education programs delivered in clinical, community, school or research settings. Further research will establish Cook-Ed^TM^’s utility in enhancing program development and in improving food and cooking skills, dietary patterns and health outcomes.

## 1. Introduction

There is a paucity of empirical research regarding which theoretical constructs contribute to producing domestic cooking programs effective in modifying dietary behaviours and diet-related health outcomes [1,2]. Few contemporary domestic cooking and food skill interventions specify the theoretical basis for the intervention, with Social Cognitive Theory being the most commonly reported behaviour change theory used [3]. Systematic reviews conclude that cooking interventions positively influence diet quality [3,4] and cooking confidence [3] with generally positive effects on other health outcomes such as blood pressure, cholesterol [4] and quality of life [3,4,5] in adults. However, these conclusions cannot be stated with certainty due to study design heterogeneity (e.g., populations studied, outcome measures), limited use of validated instruments to measure outcomes and methodological weaknesses (e.g., limited use of theory to guide content selection and evaluation, lack of control groups or power calculations, inadequate process evaluation) [3,4,6]. In a systematic review of the impact of home cooking interventions, Reicks et al. [3] highlights the need for the full integration of a theoretical framework to increase program success and strengthen evaluation.

Poor dietary patterns and excessive energy intakes are key risk factors contributing to the current chronic disease epidemic [7]. Many countries have published qualitative or quantitative food-based guidelines (FBG) to promote health and lower chronic disease risk [8]. FBG typically emphasizes the consumption of nutrient-dense whole and minimally processed foods, which often require some preparation and/or cooking to improve palatability, enjoyment, and to reduce the risk of foodborne illness. Cross-sectional studies indicate that having higher skill levels for food preparation and cooking techniques, and/or higher frequency of home cooking are associated with higher diet quality [9,10,11,12]. However, the association between cooking frequency and diet quality is not consistent across socioeconomic groups, challenging the notion that simply cooking more leads to improved diet and health outcomes [12]. Being female [13,14], older age [13,14], and having higher educational attainment [14,15] are all characteristics associated with higher cooking skill and food skill confidence. The Food Agency paradigm recognises the complex array of personal experiences, cognitive and physical capacities that interact in various social and cultural contexts to influence meal planning, preparation and cooking [16]. This highlights the need for a contemporary exploration of cooking practices, skill levels, barriers, attitudes and beliefs towards domestic and healthy cooking in the context of designing curricula to enhance cooking skills, for the purpose of improving nutrition and diet-related health. This may be particularly pertinent when developing programs for people with a socioeconomic disadvantage.

Cooking skills are primarily learned from the mother [15,17] with acquisition of cooking skills at younger ages associated with positive cooking-related behaviours and higher diet quality later in life [17]. However, it has been reported that children today are not involved in domestic cooking as frequently as they were a generation ago [10]. Wolfson et al. [15] reported that skills are commonly acquired by individuals teaching themselves, with the cooking skills taught within the home not necessarily the skills required for contemporary everyday cooking or for good health [15]. Historically, cooking skills were taught to children and adolescents via home economics classes, often in a school environment. Despite initial evidence reporting lasting effects of home economics’ education on food knowledge into adulthood [18], home economics education has largely been removed from curricula in the USA [19] and United Kingdom [20]. In Australia [21] and Ireland [20], the inconsistent inclusion of home economics curricula has been reported. Conversely, interventions to teach cooking skills as a strategy to improve diet and health have been increasingly reported in settings outside of schools [3].

Cooking education programs occur in a range of settings [3,4,6] from large-scale federally funded community cooking programs for low income populations, such as the Supplemental Nutrition Assistance Program–Education (SNAP-Ed), Cooking Matters, [22] and the Expanded Food and Nutrition Education program (EFNEP) in the USA [23], to small-scale community programs and pilot research studies [24,25,26]. Interventions can be tailored to a range of populations such as low socioeconomic groups, people affected by chronic diseases, parental status, and/or various community, religious, cultural or ethnic groups, and then grouped further based on age and/or gender. Sometimes a combination of factors (e.g., Hispanic breast cancer patients) have been used in the selection of a specific target audience [3,4,6]. Cooking programs typically comprise four to twelve weekly, fortnightly or monthly sessions, but have varied from one 20-minute session to weekly 90-minute classes, during a school term or for two and a half years [3,4,6]. The finite time available for programs to teach healthy cooking skills to a class of individuals, who inevitably have different intrinsic and extrinsic characteristics, highlights the utility of an evidence-based model to guide cooking program planning, implementation and evaluation to be cost effective in maximising learning and health-related outcomes.

Health education program developers use health promotion models and planning frameworks to ensure program design, implementation and evaluation utilise commonly accepted health behaviour change theories [27]. However, there is a paucity of models to guide healthy cooking education program planning. Raber et al. [28] describe the development of an evidence-based conceptual framework for healthy cooking behaviours associated with chronic disease prevention. However, cooking behaviour is only one aspect of the array of personal and environmental factors influencing healthy domestic food provision [16,29]. There are currently no comprehensive models available to specifically guide healthy cooking program developers in community, clinical, education or research settings.

Given that the prevalence of diet-related chronic disease is escalating rapidly worldwide, the link between cooking and food skills with diet quality and rising interest of cooking education programs, there is a need to promote evidence-based teaching of cooking and food skills. Of particular importance are programs that promote alignment with food-based guidelines and address specific nutrition needs of the individuals for whom they are developed, while also understanding the contextual factors and barriers that the target population faces. Therefore, the aim of this paper is to describe the development of the Cooking Education (“Cook-Ed^TM^”) model for planning, implementing and evaluating domestic food and cooking skill education programs that aim to improve diet and health outcomes. This paper will identify key considerations for curricula development when prioritising cooking education components within finite program schedules in different countries, settings and target groups. One scenario for applying the model is presented.

## 2. Materials and Methods

### Overview of the Construction of the Cook-Ed^TM^ Model

Three systematic reviews [3,4,6] were identified that reported on a total of 70 individual studies focused on interventions to improve cooking skills in children and adults. These reviews were read in detail to identify the methods used to design, implement and evaluate interventions.

The PRECEDE-PROCEED model is a comprehensive, health program planning and evaluation model that originated in the 1970s and has been widely applied internationally [30]. Gielen et al. [30] note that, rather than being a theory used to predict and describe why interventions work, the PRECEDE-PROCEED model provides a framework on which different health behaviour change theories can be applied. The PRECEDE- PROCEED model was selected as the basis for the Cook-Ed^TM^ model as its fundamental aspects allows the consideration of multidimensional influences on cooking behaviour which, in turn, informs targeted program development and evaluation. The PRECEDE-PROCEED model can be used to apply different behaviour change theories, allowing the integration of the theories most suitable to the target population and situation [30], and therefore presents an ideal base for a cooking education model that aims to improve dietary behaviour.

**Figure 1:**
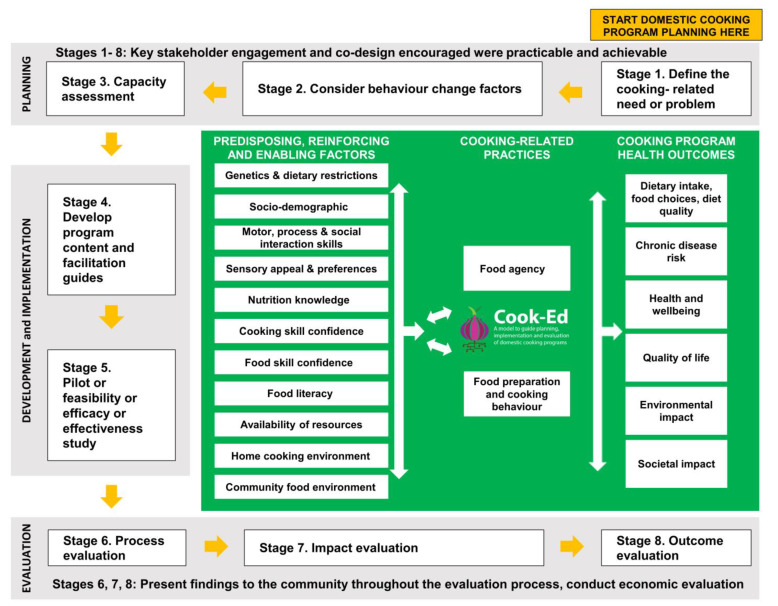
Proposed Cook-Ed^TM^ model to guide planning, implementation and evaluation of cooking education curricula for domestic cooking programs to improve diet and health.

The eight-stage Cook-Ed^TM^ model (Figure 1) was constructed by the authors RA, VS and CC based on components of the PRECEDE-PROCEED model and learnings from an examination of the current domestic cooking education literature. In 2018, an international team of co-authors with expertise in cooking and food skill education development, delivery and research was formed to review the model for content clarity. The team comprised dietitians (RA, VS, TJ, KD, TBur, CC), a public health nutritionist (FL), a public health expert (JW), consumer scientists (TB, MD), professional chefs (RA, JW), home economics educator (TJ), and an occupational therapist (AR). All co-authors provided comment throughout the iterative construction of the model until consensus on the final model was reached.

## 3. Results

### 3.1. Cook-Ed^TM^ Model Scope and Application Considerations

The Cook-Ed^TM^ model is not intended to be prescriptive, but rather components could be adopted pragmatically by cooking education program developers in community, clinical or research settings depending on the availability of resources and what is practical and achievable.

The Cook-Ed^TM^ model can be used to guide the development of cooking programs that focus on skills for preparing healthy foods and used to revise or evaluate existing programs or appraise methods used by other programs. When developing or reviewing existing cooking programs, a targeted search of the published and grey literature will help to identify previous programs used in a specific target group or setting. This can help with evaluating the existing strengths and challenges of similar programs. Potentially, this process will also identify programs that could be adapted to meet specific population needs, rather than starting from scratch.

### 3.2. Cook-Ed^TM^ Model Overview

The outer, numbered sections of the model contain eight distinct stages—three planning (Stages 1–3), two development and implementation (Stages 4 and 5), and three program evaluation stages (Stages 6–8).

The inner section of the Cook-Ed^TM^ model lists the factors preceding broader cooking-related health behaviour change on the left, and the health, environmental and societal outcomes that healthy domestic cooking programs ultimately aim to address on the right. Outcomes, practices and their contributing factors are initially explored in program planning Stages 1 and 2, findings then inform program development in Stage 4, and this forms the basis of program evaluation in Stages 7 and 8.

Cook-Ed^TM^ model components and each stage are described in further detail below. Detailed practical guidance to assist program developers, and a non-exhaustive list of evidence-based resources that may be used to measure each of the constructs listed in the model, are provided (Table A1).

### 3.3. Cook-Ed^TM^ Model Components and Stages

#### 3.3.1. Cook-Ed^TM^ Model—Inner Section

The cooking-related health outcomes we identified are listed in the model. While the Cook-Ed^TM^ model primarily aims to address cooking-related health outcomes, we also recognise that healthy cooking education programs may also seek to influence environmental outcomes through reduced food waste and sustainable food choices [20]. While it was not the aim, nor was it within the scope of the Cook-Ed^TM^ model to describe the methods for demonstrating societal impact, this is the ultimate goal of health promotion, thus societal impact is also factored in the model.

Cooking-related practices listed in the Cook-Ed^TM^ model, Food Agency and food preparation and cooking behaviours, are the identified proximal factors that influence cooking-related health outcomes. As established in the cooking literature, these proximal factors can be influenced by the interaction of a number of predisposing, reinforcing and enabling factors. Some factors, such as skills and knowledge, are modifiable, while others, such as dietary restrictions, will not be modifiable, or some are not modifiable within the scope of the program.

#### 3.3.2. Cook-Ed^TM^ Model—Planning, Stages 1–3

The Cook-Ed^TM^ model planning phase examines the cooking-related health problem or need, its preceding or contributing factors, and the available resources and capacity to develop and deliver the program.

Stage 1—Define the cooking-related need or problem and, engage key stakeholders using co-design principles in all stages:

- Conduct social, epidemiologic and ecologic assessment to analyse gaps in the current knowledge, the need or problem, and to gain an understanding of the community and the specific nutrition and/or health issues to be addressed;
- Identify and engage with key stakeholder at the outset using a consultative approach, based on co-design principles of equal partnerships, openness, respect, empathy and designing together, at every stage [31]. Reflect and share experiences and findings with community partners, key stakeholders and other program developers alike to support program sustainability and inform future programs [32].

Stage 2—Consider behaviour change factors:

- Identify, prioritise and examine the cooking-related practices and the predisposing, reinforcing and enabling factors that influence the need or problem identified in Stage 1.

Stage 3—Capacity assessment:

- Consider the economic, personnel and physical resources available to run the cooking program in the short term, and program sustainability;
- Investigate health and safety considerations (e.g., completion of safe food handling courses, police and child protection checks for programs involving minors) and local food regulations;
- Where necessary, conduct risk assessment and obtain human research ethics approval.

#### 3.3.3. Cook-Ed^TM^ Model–Development and Implementation, Stages 4–5

In the Cook-Ed^TM^ model development phase, the findings from the planning phase are used to develop and test program content that is tailored to the needs, preferences, skills and resources of the target population. Evaluation data from earlier programs, feasibility studies or pilot data can be used at this stage to iteratively develop or modify programs.

Stage 4—Develop program content and facilitation guides:

- Define program aims to address the health issues identified in Stage 1;
- Define program objectives to address the cooking-related practices and predisposing, reinforcing and enabling factors identified in Stage 2;
- Align content with program aims, objectives and capacity assessment (Stage 3);
- To promote the development of cooking and food skills of highest priority, consider relevant food-based guidelines when selecting recipes and determining program content;
- Select program format such as face-to-face, demonstration, hands-on and/or virtual delivery;
- Select a pedagogy, or behaviour change strategies to guide the teaching and the development of learning resources. Consider using a participant-centred education approach such as Experiential Learning, as opposed to a teacher-centred approach;
- If required, engage an Occupational Therapist (OT) in a consultative capacity in program design;
- Consider building in provisions to allow facilitators to tailor the program to the needs of individuals and/or groups, depending on the demands of the activities on individuals, whether activities need modification, or what supports (e.g., physical, verbal and/or visual cues, or assistive technology (AT)) may be required;
- An OT can complete comprehensive assessments with individuals to inform the use of adapted activities, graded assistance, and enable access to AT. Develop processes to enable timely referral to an OT for assessment if needed;
- Develop and select process, impact and outcome evaluation tools and measures (Table A1).
- Stage 5—Pilot or feasibility or efficacy or effectiveness study:
- Pilot the program during development to test recruitment strategies, program content and resources, and evaluation tools and methods;
- Feasibility may be enhanced by adopting an iterative process based on feedback and evaluation (Stages 6–8).

#### 3.3.4. Cook-Ed^TM^ Model—Evaluation

Stages 6–8

The Cook-Ed^TM^ model evaluation stages gather information on program delivery, short and long-term program effectiveness. This can strengthen the evidence-base for domestic cooking education as a strategy to improve diet and health.

Stage 6—Conduct process evaluation:

- Conduct process evaluation throughout, and post-program implementation to examine recruitment, participant retention and exposure to the intervention, and to assess the degree to which the program was implemented as intended (i.e., program fidelity);
- Process evaluation instruments can be individualised to unique cooking programs.

Stage 7 and 8—Conduct impact and outcome evaluation:

- Conduct impact and outcome evaluation pre- and post-program implementation;
- Impact evaluation examines the predisposing, reinforcing and enabling factors (identified in Stage 2) which the program aimed to modify, and how these influenced the cooking-related practices of participants;
- Outcome evaluation measures refer to the broader long-term health outcomes. Align outcome evaluation measures with program aims, and that address the cooking-related need or problem identified in Stage 1;
- Where available, use validated instruments to conduct impact and outcome evaluation (Table A1).

In addition to process, impact and outcome evaluation, examine capacity gain and conduct economic evaluation to provide information on sustainability and efficiency, respectively [32]. Disseminate evaluation findings to key stakeholders. Ensure the format that information and findings are presented in is accessible to all stakeholders.

In the next section, a scenario for developing a specific cooking education program is described to illustrate how the Cook-Ed^TM^ model can be applied in practice.

### 3.4. Scenario—A Cooking Intervention for Young Adults with Intellectual Disability

Background: A disability support provider identifies a need for, and interest in, a healthy home cooking education program for their young adult clients with mild–moderate intellectual disability. Intellectual disability can be classified depending on severity. When given the right support, people with mild-moderate intellectual disability can acquire the skills needed for relatively independent living, compared to more severe forms of intellectual disability which may require considerably greater support and supervision [33]. A partnership between the service provider and a local university nutrition team was formed to co-design, develop, deliver and evaluate the program. A representative from the intellectual disability community is recruited to provide further input into program development. The published literature and data collected from a representative sample of the intellectual disability community and program participants, before and after program implementation, respectively, is used to inform program development and ongoing program improvement. Further detail is provided in Table 1.

## 4. Discussion

The Cook-Ed^TM^ model for planning, implementing and evaluating cooking education programs was informed by: the existing evidence-base for cooking education; the PRECEDE-PROCEED model for health promotion; other public health nutrition program planning, implementation and evaluation models; experiential learning and the consensus of our international team who have expertise in cooking and food skill education programs or research studies. The Cook-Ed^TM^ model identifies candidate factors for evaluation in pre-program planning to inform content development and post-program evaluation. This can be used to refine cooking education programs and help to advance the evidence base for healthy cooking programs’ effectiveness in improving dietary behaviour and diet-related health outcomes. The scenario presented demonstrates how Cook-Ed^TM^ can be applied to develop a new cooking education program.

Cooking education programs can be resource-intensive, requiring suitably qualified staff to develop and implement curricula, recruit participants and evaluate the cooking program outcomes. Considering which programs exist in the community and forming partnerships with organisations can be useful to achieve efficient and economic program development and delivery [35]. Adopting co-design principles from the planning stage has the potential to assist in the development of tailored programs targeted to the needs of end users [31,36], support meaningful, effective, committed partnerships, and promote program success [30]. Additional resource requirements include the availability of a safe and accessible learning environment that is suitable and equipped for food preparation. However, rather than relying on the availability of fully equipped kitchens, innovative methods may be used to convert rooms into suitable cooking spaces [24], while virtual program delivery has the potential to reach large numbers of participants globally [37].

A number of personal and environmental factors influencing healthy domestic cooking practices were identified during the development of the Cook-Ed^TM^ model. Cooking program developers should consider those most relevant and applicable to their target population. By examining sociodemographic factors, programs that are culturally, socioeconomically and age appropriate can be developed. Cooking confidence, food skill confidence and self-efficacy in one’s abilities is associated with the increased use of basic ingredients and increased willingness to experiment with different foods, having a greater repertoire of meals to prepare, and healthier food choices [9,38,39,40]. Furthermore, limited nutrition knowledge may act as a barrier to healthy food choices [41] and further influence cooking skills (e.g., cooking method selection) and food skills (e.g., shopping and meal planning). The capacity to safely learn and practise particular cooking skills may vary according to the age of the population, cognitive and physical capacity or presence of intellectual or physical disabilities [42]. While a particular diagnosis may be a clear indicator, at times, individuals may have more subtle underlying issues that emerge within a complex task such as cooking. It is essential that these are identified and considered in order to facilitate positive experiences, provide the right level of challenge, encourage ongoing engagement and help to ensure participant safety [43].

Limited access to utensils for cooking and suitable areas for food preparation and storage in the home are other factors that may influence cooking behaviour [29,44]. The influence of the community food environment (e.g., access to transport, grocery stores and variety of food stocked) on dietary patterns has been well documented [45]. By considering the availability of resources, such as the time needed to procure, prepare and cook food, support or supervision available for particular sub-groups, such as children, and the availability of space, utensils and appliances to store, prepare and cook food (i.e., home cooking environment and equipment), content that supports the development of skills which are transferrable from the cooking program to participants’ domestic kitchen can be created. Food literacy is a comprehensive set of interrelated skills and knowledge needed for meal provision, food preparation and cooking, influenced by different environmental contexts [29]. Wolfson et al. [2] highlights that interventions based on food-agency have the potential to support program recipients with the technical skills of cooking and knowledge of nutrition, while developing the skills required to overcome social and environmental challenges or barriers. Finally, the enjoyment of cooking is an important determinant of whether people actually implement and prioritise home cooking in their daily lives [46]. Establishing what the target population wants to cook, the sensory appeal of food, skills they perceive they want to acquire or improve, and any dietary restrictions, will guide the selection of enjoyable activities that promote engagement.

Program developers should select a pedagogy to guide teaching and the development of learning resources. Hollywood et al. [1] notes that there is a paucity of learning theory applied or explicitly stated in cooking interventions. Experiential Learning Theory (ELT) is a participant-centred education approach that recognises that learning is an adaptive process occurring across human settings, encompassing all life stages [47,48]. ELT views the outcome of learning as the development of skills and knowledge for engaging in a lifelong learning process, rather than the accumulation of factual knowledge. ELT values interactions between program coordinators and participants as an essential component in the learning process and considers experience, perception, cognition and behaviour in learning, thus recognising the different learning styles held by individuals [47,48].

Cooking education programs may include hands-on or demonstration-based cooking instruction or a combination. Wolfson et al. [2] proposes that repetitive, hands-on cooking skill development is a fundamental aspect to the development of food agency. There is some evidence to suggest that hands-on cooking classes, rather than demonstrations, are more beneficial in positively affecting behaviour change, cooking attitude and self-efficacy in sophomore-level college students [49]. However, this was the only published study identified that directly compared the two methods. Hollywood et al. [1] notes cooking and food skill interventions utilising practical behaviour change techniques, rather than demonstration, were more beneficial in promoting health behaviour change. This was particularly evident in studies reporting longer-term (greater than 3 months) behaviour change [1].

A frequently reported limitation of existing cooking interventions is the limited use of high-quality, validated instruments to assess the factors influencing healthy cooking behaviour and program outcomes [3,4,9], however, validated instruments are emerging (Table A1). Few long-term impacts of cooking interventions are reported [1]. For future research, studies’ sample sizes should be calculated ‘a priori’. The use of control or comparison groups in evaluation of cooking studies has been limited to date, and is recommended for future programs [3,4]. In research evaluating cooking interventions, strong study design, and rigorous analysis and reporting of process, impact and outcome evaluation, would assist future cooking curricula developers and build a higher quality evidence base in this field [3,4].

A limitation of the Cook-Ed^TM^ model is that the evidence of what contributes to cooking interventions’ effectiveness in improving diet-related behaviour and health outcomes is inconsistent. Tools to evaluate cooking education programs are, at present, restricted to only a few validated tools that we are aware of. Cooking education for improving diet-related health is an important but emerging area of research, which would benefit from more robust research, including rigorous study designs and the development of validated tools for use in program evaluation.

## 5. Conclusions

The current paper presents a number of factors for consideration when planning, implementing and evaluating cooking education programs and maps an existing intervention against the evidence base. Further research is required to establish the Cook-Ed^TM^ model’s effectiveness in improving cooking-related health outcomes of real-world cooking education programs. We recommend that future research studies use the model to guide the planning, implementation and evaluation of cooking education programs to facilitate future refinement to the model and its implementation. Reporting should clearly identify whether the Cook-Ed^TM^ model was used and how each component of the model was applied, or not, to enable the utility and effectiveness of the Cook-Ed^TM^ model to be evaluated in differing research and community health settings and population groups.

## Figures and Tables

**Table 1:** Example of Cook-Ed^TM^ model activities in the development of a cooking intervention for young adults with mild–moderate intellectual disability.

**Cook-Ed^TM^ Stage**	**Activity**	**Description**
**Planning**		
Stage 1: Define the cooking-related need or problem and engage key stakeholders using co-design principles in all stages	Review the published literature and health data; Engage stakeholders and community partners; Prepare ethics documents.	Increased rates of diet and lifestyle-related modifiable chronic disease risk and poorer quality of life in people with intellectual disability, compared to peers without disability, is confirmed through review of the published literature. Findings are used to inform program aims; Co-designed surveys, interview and focus group protocols are developed to establish a deeper understanding of the health-related outcomes identified, and contributing cooking-related behaviour change factors (Stage 2); Institutional ethics approval is obtained to gather, evaluate and publish data that will inform program content and evaluation.
Stage 2: Consider behaviour change factors	Surveys and focus groups with key stakeholders	Hypothesised behaviour change factors related to cooking behaviour in this population included motor and process skills, preferences and dietary restrictions, cooking and food skills, and home cooking environment; Young adults with intellectual disability (i.e., potential participants) and carers and family members, and support workers are invited to participate in the co-designed interviews, focus groups and surveys. Findings are used to align program objectives with end users’ needs.
Stage 3: Capacity Assessment	Assess available resources; Seek required resources; Conduct risk assessment; Consider relevant policy and regulations.	An existing work health and safety compliant, accessible teaching kitchen within the university is located; Given the unique and individual challenges presented with intellectual disability, expertise in Occupational Therapy (OT) is sought through the university; Personnel to assist with program evaluation are identified as an essential resource, and sought through Nutrition and Dietetic and OT student volunteer registers; Risk assessment is conducted and institutional safety clearances obtained; Institutional ethics approval is obtained to deliver and evaluate a feasibility study of the cooking program.
**Development and Implementation**		
Stage 4: Develop program content and facilitation guides	Define program aims and objectives; Develop program content and teaching resources; Select program evaluation tools.	Content is developed to address aims and objectives, based on findings in Stage 1 and 2, and with consideration to the resources identified in Stage 3 (e.g., the size of each group for education session was limited to maximum 6 participants); Standard program content is developed with provisions for content to be adapted (e.g., additional recipes, recipe template to create new/tailored recipes) to suit the individual needs and priorities of each group; Few validated tools to assess outcomes in this population are identified, hence existing tools are modified, informed by literature review findings in Stage 1 and 2 and through consultation with key stakeholders and experts in the field of disability education and research; Resources were not available to validate these tools for the specific target group prior to program delivery, thus validation activities are planned pragmatically throughout program implementation.; Accessible [34] process evaluation tools specific to the study are developed; Feedback on the suitability and relevance of program content, design and evaluation tools is provided by a potential participant with a mild intellectual disability who is recruited to the research team as a consumer representative and paid a consultation fee.
Stage 5: Pilot or feasibility or efficacy or effectiveness study	Pre-pilot	A small pre-pilot is conducted with eligible participants (with support workers present) to test program resources and evaluation tools before the full program is commenced.
**Evaluation**		
Stage 6: Conduct process evaluation Stage 7: Conduct impact evaluationStage 8: Conduct outcome evaluation	Collect baseline data; Collect process evaluation data at each session and at the final session; Collect impact and outcome evaluation data post-program and follow up; Conduct economic evaluation; Present findings; Seek stakeholder and community partner feedback	On cooking program enrolment, participants complete a modified, accessible [34] survey to assess preferences and restrictions and home cooking environment (with support worker assistance), to enable the prioritisation and tailoring of program content. *Process evaluation* During program implementation, the facilitator records session length, attendance, activity implementation and participation, to inform program feasibility and fidelity assessment; On program completion, participants complete accessible [34] participant satisfaction surveys to inform process evaluation and program acceptability. *Impact and outcome evaluation* On program commencement, program participants complete surveys to evaluate the health outcomes identified in Stage 1 (i.e., dietary intake and quality of life) and hypothesized behaviour change factors the program sought to address (i.e., motor and process skills, cooking and food skills confidence). These are again completed upon program completion and at 6- and 12-months post-program, providing data for impact and outcome evaluation; Throughout the process (Stage 1–Stage 8), program costs are recorded for economic evaluation; Process and impact evaluation data, economic evaluation, and stakeholder and community feedback is used to inform each new iteration of the program.

## Appendix A

**Table A1:** An inventory of resources to assist cooking program developers.

**Construct**	**Tool/Source**
*Stage 1: Define the cooking-related need or problem*	
Human Research Ethics	See research ethics committee relevant to your institution and/or jurisdiction
**Inclusive/participatory research**	
Consumer and community involvement in research	Statement on consumer and community involvement in health and medical research [50]
**Dietary Intake, food choices and diet quality**	
Dietary intake ^1^	Australian Recommended Food Score (ARFS) [51]
Fruit and vegetable variety ^1^	Fruit And Vegetable VAriety index (FAVVA) [52]
*Stage 2: Consider behaviour change factors (cooking-related practices and predisposing, reinforcing, enabling factors)* *Also useful in Stages 6, 7, 8–process, impact and outcome evaluation*	
**Sociodemographic factors**	
Sociodemographic survey questions (Australia)	Australian Bureau of Statistics 2016 census questions [53]
Socioeconomic disadvantage (Australia)	Socioeconomic Indexes for Area [54]
**Motor and process skills**	
Shopping, community access, and meal preparation ^1^ Independence, safety and adequacy in tasks for community living ^1^	Occupational analysis via non-standardized assessment [55] Performance assessment of Self-care skills (PASS) [56]
Motor and process skills ^1^ Note: OT must be trained to use the AMPS.	Assessment motor and process skills (AMPS) [57,58]
**Food preferences, dietary restrictions and sensory appeal**	
Food choice ^1^	Food choice questionnaire [59]
Food choice ^1^	Food choice and applied nutrition [60]
Health, taste and attitudes ^1^	The Health Taste and Attitude Scale [61]
**Nutrition knowledge**	
Nutrition knowledge—short ^1^	PKB-7 scale [62]
Nutrition knowledge—comprehensive ^1^	Re-examined General Nutrition Knowledge Questionnaire (GNKQ-R) [63]
Nutrition knowledge—Australian version ^1^	Revised General Nutrition Knowledge Questionnaire for Australia [64]
**Cooking and food skills**	
Cooking and food skill confidence ^1^	Cooking and food skill confidence in adults [65] and children
Food skills acquisition ^1^	An evaluation tool for measuring food skill acquisition [66]
Perceived cooking competence – children^1^	Children’s perceived cooking competence measure [67]
**Food literacy**	
Evaluation of food literacy program ^1^	Evaluation Tool Development for Food Literacy Programs [68]
**Food agency ^1^**	Cooking and food provisioning action scale CAFPAS [69]
**Food preparation and cooking behaviour**	
Orientation toward food preparation ^1^	Food involvement scale [70]
**Home cooking environment**	Home CookERI^TM^ [71]
*Stage 3: Capacity Assessment*	
Program sustainability	Program Sustainability Assessment Tool (PSAT) [72]
*Stage 4: Develop Program*	
Selection of behaviour change techniques	The CALO-RE taxonomy [73]
Selection of cooking curricular	Evidence based framework for healthy cooking [28]
*Stage 5–8: Evaluation*	
Translational research assessment	Translational Research Framework [74,75]

^1^ Data collected using these instruments can be used to inform program planning and content development, and also in pre- and post-program evaluations and to inform ongoing program improvement.

Published research or relevant local, state or national population data should be used to inform Stage 1. Information may be collected from the target audience or the published literature. Where cooking interventions target a specific population subset, and published data for the target group are absent, program developers can collect data from a representative sample. This could be combined with an assessment of cooking-related practices and their predisposing, reinforcing and enabling factors for efficiency and to minimise participant burden. Community advisory groups and key stakeholder forums at this stage, as with all stages of the Cook-Ed^TM^ model, are suggested. Data collected for research purposes require relevant human research ethics clearance and participant consent. Depending on the jurisdiction, approval may also be required for quality assurance projects. The health outcomes, cooking-related practices and their predisposing, reinforcing and enabling factors we have identified are located in the centre section of the Cook-Ed^TM^ model, and are described in more detail below.

**Dietary Intake, food choices and diet quality:** Evaluations to date mainly utilise quantitative methods such as 24-h recall and food frequency questionnaires [3,4,6]. Image-based methods are also emerging [76]. Outcomes can be analysed and reported as changes in energy intake, individual nutrients (e.g., sodium, saturated fat) and/or intake of individual food groups, with fruit and/or vegetable being the most commonly reported [3,4,6]. Consumption of ready meals, convenience food and/or food consumed outside of the home may also be reported [3,4,12].

**Sociodemographic characteristics:** Consider cultural background, information about whom participants usually cook for or with, household food budget, food access, literacy and highest education level. Data collected prior to implementing a program can be used by program developers to enhance the capacity to personalise cooking program content so that the curriculum builds on participants’ existing skills and abilities. When reviewing data/evaluating processes issues such as sociodemographic factors need to be considered to reduce the confounding of empirical data collected. While recruitment for cooking programs will generally target a particular age group, a brief paper-based or online questionnaire, or discussion with participants prior to commencing the program, would identify key data on relevant factors to allow content for each session/program to be modified to suit the group. In some cases, it may not be possible to collect the information from program participants themselves, thus requiring collection from others on behalf of participants. Examples of this may include those with impaired cognition, where family, carers or support workers may provide or assist in the collection of information. In the case of children, this information will often be collected from a parent or guardian.

**Motor, process and social interaction skills:** Where difficulties in performance are noted, enabling access to further assessment and intervention may promote engagement in cooking and other Independent Activities of Daily Living (IADL). Consultation with an Occupational Therapist (OT) in such cases can inform tailored supports for clients, instructors and program developers. OT assessments range from non-standardised observation of the client in their home environment using activity analysis skills, to standardised assessments that assist OTs in determining the specific person, task, and/or environment factors contributing to occupational performance discrepancy. Standardised assessments such as the Performance Assessment of Self Care Skills (PASS) [56] Assessment of Motor and Processing Skills (AMPS) [57,58] and can inform tailored strategies to improve performance in IADL, such as shopping and cooking skills. Strategies found to be beneficial for those with impaired executive function include environmental cognitive supports such as signs, checklists and other compensatory strategies [77]. Assistive technology (AT) can increase client engagement and safety in meal preparation and includes items such as electric can openers, built-up handles, devices which emit sound and Apps. While AT can be implemented independently by participants and program coordinators in certain cases, additional assessment may be required in order to support appropriate prescription that ensures the safety, cost effectiveness and use of AT.

**Food preferences dietary restrictions and sensory appeal:** Program developers should incorporate assessment of food preferences, dietary restrictions and sensory appeal into a pre-program registration/questionnaire and adapt program content accordingly. Consideration of typical human development across the lifespan is necessary in ensuring that program content is relevant to the everyday cooking needs of the target population [78]. Facilitating choice is an important consideration across the lifespan. However, at key stages such as young adulthood, when individuals develop a greater sense of self and become increasingly independent, enabling opportunities for greater autonomy is an essential consideration [42]. Be aware if the individual or the people they cook for have dietary restrictions that need to be catered for in home cooking, e.g., the elimination of gluten (coeliac disease) or diagnosed food allergies.

**Cooking and food skill confidence:** Cooking skills refer to a range of food preparation and cooking techniques, both physical and mechanical such as chopping, mixing and heating [65,79,80]. Food skills are a distinct set of non-cooking skills that enable individuals to apply knowledge about food to prepare meals and snacks that are nutritionally appropriate with the available resources. Food skills include meal planning, budgeting, shopping, resourcefulness and label reading [65,81]. Program developers may draw upon existing data or pre-program planning activities such as surveys or interviews and the course of program evaluation. Individualised data may be collected at the commencement of the program.

**Food preparation and cooking behaviour:** Evaluation of food preparation and cooking behaviours may include: frequency of cooking with basic or fresh foods [37,82] or food/beverage consumed at home [11,37]; frequency eating prepared meals such as ready-meals or take-away/fast food [11,82].

**Determine priority cooking skills to include in a program:** Select recipes to promote the development of skills considered relevant or high-priority to improve diet quality, based on the number of servings from each food group by sex and age and dietary intake data of the target population. A generic cooking intervention for Australian children and adolescents (aged 4–18 years) would have ~30% of learning time focused on skills associated with vegetable intake, ~30% on grains, ~15% on dairy and alternatives, ~15% on meats/poultry/fish/eggs/tofu/nuts/seeds/legumes/beans, and ~10% on fruit. If data indicates that program participants are not meeting FBGs for specific food groups, then a greater proportion of time could be allocated to skills needed to prepare those foods. For example, looking at national dietary intake data in Australia [83], it could be justified to direct greater attention to preparing vegetables/legumes (all age/sex groups), whole grains (all age/sex groups) and, for some groups, ‘dairy’ or iron in females age 14–18 years or a reduction in EDNP foods for everyone, but particularly adolescents. Recipes should be selected to promote the development of skills considered relevant or high-priority and align with overall program aims to improve diet quality based on the number of servings from each food group by sex and age and dietary intake data of the target population. Collaborative development of a ‘kitchen agreement’ to guide safe, respectful and appropriate behaviours from group members can be useful to optimise safety, group harmony and positive outcomes for the program. The Raber et al. [28] framework comprises five individual constructs—cooking frequency, techniques and methods, minimal usage, flavouring, and ingredient additions/replacements—and can be useful when selecting, and/or modifying recipes and educational content.
